# Impact of collaborative physician-pharmacist stewardship strategies on prophylactic antibiotic practices: a quasi-experimental study

**DOI:** 10.1186/s13756-022-01138-3

**Published:** 2022-07-26

**Authors:** Antonelle Pardo, Vianney Ntabaza, Mathieu Rivolta, Aline Goulard, Serge Sténuit, Remy Demeester, Sandrine Milas, Pierre Duez, Stéphanie Patris, Marc Joris, Philippe Dony, Soraya Cherifi

**Affiliations:** 1 Laboratory of Therapeutic Chemistry and Pharmacognosy, Faculty of Medicine and Pharmacy, University of Mons, Mons, Belgium; 2 Department of Clinical Pharmacy, Faculty of Medicine and Pharmacy, University of Mons, Mons, Belgium; 3 Laboratory of Pharmacognosy, Faculty of Pharmaceutical Sciences, University of Lubumbashi, Lubumbashi, Democratic Republic of Congo; 4 Mathematics Department, Faculty of Sciences, University of Mons, Mons, Belgium; 5 Department of Pharmacy, University Hospital of Charleroi, Charleroi, Belgium; 6 Department of Internal Medicine and Infectious Diseases, University Hospital of Charleroi, Charleroi, Belgium; 7 Department of Cardiothoracic Surgery, University Hospital of Charleroi, Charleroi, Belgium; 8 Department of Anesthesiology, University Hospital of Charleroi, Charleroi, Belgium

**Keywords:** Pharmacist, Antibiotic prophylaxis, Surgery, Infections, Stewardship

## Abstract

**Background:**

An effective use of surgical antibiotic prophylaxis (SAP) appears essential to prevent the development of infections linked to surgery while inappropriate and excessive prescriptions of prophylactic antibiotics increase the risk of adverse effects, bacterial resistance and *Clostridium difficile* infections. In this study, we aimed to analyze SAP practices in an acute secondary hospital in Belgium during the years 2016–2021 in order to evaluate the impacts of combined stewardship interventions, implemented thanks to a physician-pharmacist collaboration.

**Methods:**

A quasi-experimental study on SAP practices was conducted during 5 years (2016–2021) in a Belgian University Hospital. We first performed a retrospective observational transversal study on a baseline group (2016.1–2016.4). Then, we constituted a group of patients (2017.1–2017.4) to test a combined intervention strategy of stewardship which integrated the central role of a pharmacist in antibiotic stewardship team and in the pre-operative delivery of nominative kits of antibiotics adapted to patient factors. After this test, we collected patient data (2018.1–2018.4) to evaluate the sustained effects of stewardship interventions. Furthermore, we evaluated SAP practices (2019.1–2019.4) after the diffusion of a computerized decision support system. Finally, we analyzed SAP practices in the context of the COVID-19 pandemic (2020.1–2020.4 and 2021.1–2021.4). The groups were compared from year to year in terms of compliance to institutional guidelines, as evaluated from seven criteria (χ2 test).

**Results:**

In total, 760 surgical interventions were recorded. The observational study within the baseline group showed that true penicillin allergy, certain types of surgery and certain practitioners were associated with non-compliance (*p* < 0.05). Compared with the baseline group, the compliance was significantly increased in the test group for all seven criteria assessed (*p* < 0.05). However, the effects were not fully sustained after discontinuation of the active interventions. Following the diffusion of the computerized decision support system, the compliance to guidelines was not significantly improved. Finally, the COVID-19 pandemic did not appear to affect the practices in terms of compliance to guidelines.

**Conclusions:**

This study shows that optimization of SAP practices is achievable within a proactive multidisciplinary approach including real-time pharmaceutical interventions in the operating area and in the care units practicing SAP.

## Background

The Lancet Commission on Global Surgery identified that 313 million surgical procedures are performed worldwide each year and that at least 4.2 million people worldwide die within 30 days of surgery each year. This number of postoperative deaths accounts for 7.7% of all deaths globally, making it the third greatest contributor to deaths in the world [1]. In a study of the University Hospital of Charleroi in Belgium, post-operative mortality rates within 30 post-operative days were 1.1% [2]. In this hospital, risk factors of post-operative mortality were identified as the absence of an anesthesia nurse, American Society of Anesthesiologists scores (ASA scores) > 2, emergency, duration of surgery and rate of admission to critical care unit [2, 3].

Surgical site infections (SSIs) have been shown to compose up to 20% of all healthcare-associated infections [5]. Appearing in at least 5% of patients undergoing a surgical procedure, SSIs are an important source of morbidity which increase the postoperative mortality [4, 5]. The use of surgical antibiotic prophylaxis (SAP) is an effective measure to prevent the development of SSIs. In addition, it appears that inappropriate and excessive prescriptions of prophylactic antibiotics increase the risk of adverse effects, bacterial resistance and *Clostridium difficile* infections, but also increase the length of stay and the costs of health care [6–8].

To evaluate the compliance of SAP practices to guidelines, several criteria for SAP prescriptions can be observed: the indication, the antibiotic agent, the antibiotic dose, the route of administration, the timing of administration, the number of administrations and the duration of the prophylaxis [6]. In our study, we mainly focused on surgical interventions to which surveillance was recommended by the Belgian Antibiotic Policy Coordination Committee (BAPCOC): hip prosthesis, coronary artery bypass grafting, colorectal intervention and endoscopic prostate resection [9].

Recommended tools for an action plan include: (A) an antibiotic stewarship multidisciplinary team, (B) local guidelines, (C) implementation of guidelines, (D) specific prescriptions and stop orders, (E) expert systems and audits. Indeed, according to the literature, individual and educational barriers could be overcome by local consensus (tool B) and education [10]. Education is considered essential in a stewardship strategy, but studies confirm that education alone, without the incorporation of active actions, is not significantly effective in improving the frequency of compliance with practices [11–14].

Previous published papers showed that SAP practices could be optimized by the implementation of isolated strategies such as the pre-operative delivery of nominative kits of antibiotics [15], the implementation of a computer-based prescription system [16], and pharmacist Interventions [17–19].

In Belgium, Management Group of Antibiotics are regulated under Belgian law and have been mandatory since 2007 in all acute hospitals and in all large chronic hospitals (with a minimum of 150 beds). Belgian Management Group of Antibiotics should be composed of at least the following members: the hospital's antibiotic therapy management delegate, a hospital pharmacist, physicians from different specialties (clinical infectiology and/or medical microbiology, hospital hygiene), a clinical biologist who can be a physician or a pharmacist. In 2016, the Belgian University Hospital in which this study was conducted was equipped with tools (A) and (E), tool (B) having to be partly updated. To implement updated institutional guidelines (tool (C), it was necessary to solve the three types of barriers to implementation: individual, educational and structural. The structural barrier could be lifted by a structural solution such as the modification of the role of the pharmacy. During the year 2016, it was therefore decided that the updating of the institutional guidelines and the education of the practitioners applying antibiotic prophylaxis would be carried out by three members of the *Management Group of Antibiotics* of the hospital: a pharmacist, a microbiologist and an infectious disease specialist. These three members constituted an antibiotic stewardship multidisciplinary team dedicated to SAP.

In this study, we aimed to evaluate the impacts on SAP practices of several collaborative physician-pharmacist strategies implemented after the identification of risk factors associated with non-compliance towards updated institutional guidelines. SAP practices were thus studied each year from 2016 to 2021, knowing that the years 2020 and 2021 were affected by the Coronavirus 2019 disease (COVID-19) pandemic. A previous study carried out in our hospital revealed that antibiotics were overused during the COVID-19 pandemic in 2020 [20]. Therefore, our last audits aimed to evaluate the impact of the COVID-19 pandemic on SAP practices.

## Methods

### Design, setting and participants

A retrospective study was performed on a cohort of patients hospitalized in a 600-bed teaching hospital in Charleroi, Belgium. Patients were included if they were at least 18 years old and had undergone one of the following operations: hip prosthesis, coronary artery bypass grafting (CABG), colorectal surgery, transurethral resection of the prostate, endoscopic retrograde cholangiopancreaticography (ERCP). Patients were excluded if they were diagnosed as infected at the time of the surgery. Patients under 18 years old were also excluded.

Six groups of patients were thus constituted during the following time periods of 15 weeks:

- period 0: between January 11, 2016 and April 22, 2016 (baseline group)
- period 1: between January 9, 2017 and April 21, 2017 (test group)
- period 2: between January 8, 2018 and April 20, 2018 (post-test group)
- period 3: between January 7, 2019 and April 19, 2019 (post-computerized tool group)
- period 4: between January 6, 2020 and April 17, 2020 (first group of the COVID-19 period)
- period 5: between January 4, 2021 and April 16, 2021 (second group of the COVID-19 period).

The baseline group was constituted during a pre-intervention stage (period 0) without stewardship. On this baseline group, we performed an observational transversal study in order to identify risk factors associated with non-compliance towards prophylactic antibiotic guidelines.

Then, we conducted a quasi-experimental study to assess the impacts of collaborative physician-pharmacist stewardship strategies on SAP practices from 2016 to 2021. In the test period of 2017, which served as an interventional period, a full-time pharmacist provided guidance to prescribing physicians in order to optimize SAP practices. The test group was constituted during this test period (period 1). Then, the pharmacist retrospectively audited the test group in terms of SAP practices. Real-time pharmaceutical stewardship was discontinued after the period 1 and to evaluate the sustained effects of stewardship, SAP practices were also audited in a cohort of patients hospitalized in 2018 after the interventional period (post-test group). The impact of the diffusion of a computerized decision support tool was then assessed by auditing SAP practices in 2019 in the post-computerized tool group. Finally, we evaluated the impact of the COVID-19 pandemic by evaluating SAP practices on two cohorts of patients operated respectively in 2020 (first group of the COVID-19 period) and in 2021 (second group of the COVID-19 period).

### Criteria

With reference to the Belgian/Luxembourg edition of the Sanford guide to antimicrobial therapy [21] and the recommendations of several American scientific societies summarized in one report [22], the antibiotic stewardship multidisciplinary team of the hospital established in 2016 institutional guidelines which specified, for each type of intervention, the antibiotic prophylaxis regimen to use when it is indicated. These updated institutional guidelines were also based on the results of the antimicrobial resistance analysis within the hospital. These guidelines cover all surgical disciplines for adult patients within the hospital and promote the rational use of antibiotic prophylaxis starting in the pre-operative period of specific clean and clean contaminated operations.

To evaluate the use of prophylactic antibiotics in the hospital, for the five interventions audited, seven parameters (Indication, Drug agent(s), Dose(s), Route of administration, Time of pre-operative dose administration, Number of administration(s), Duration of prophylaxis) were assessed against the institutional guidelines (Table 1).

**Table 1:** Institutional Criteria for the rational use of antibiotic prophylaxis

Assessed parameter included in the institutional guidelines	Recommendations for rational use
Indication for prophylaxis	Specific interventions of clean and clean contaminated operations where the benefit is demonstrated
Recommended drug(s) (+ Alternative drugs for patients with IgE-mediated allergy to the recommended drug(s))	Antibiotics active on bacteria presumed responsible for infections (incision site/surgical site) with the narrowest spectrum of antibacterial activity. The prophylactic regimen should cover MRSA for carriers identified before the intervention
Dose of anti-infective agent(s)	Determination of the antibacterial dose by integrating the following elements: The individual characteristics of the patient: its weight and its rate of glomerular filtration (in renal impairment, the first dose does not require dose adjustment but the subsequent doses may need adjustment according to the results of glomerular filtration rates) The antimicrobial specific pharmacokinetic and pharmacodynamics properties
Number of administration(s)	Determination of the number of administration(s) by integrating the following elements: The maximum duration of prophylaxis The patient's glomerular filtration rate (in renal impairment, the first dose does not require adjustment but the number of subsequent doses may need adjustment according to the results of glomerular filtration rates) The half-life of the drug The type and the duration of the intervention and the volume of blood lost during the intervention
Route of administration	Intravenous route generally. Oral route for antibiotics that reach equivalent tissue concentration when given orally
Time of administration	The most important administration is that performed before the incision. The timing of this administration depends on the infusion time (i.v route) or the absorption time (oral route) of the drug agent: The antibiotic must be administered 15–60 min before the incision for antibiotics with rapid i.v administration (e.g. cefazolin). Earlier administration is necessary for i.v. antibiotics which must be administered over a period of ≥ 60 min (e.g. 2 h before the incision for vancomycin) or for oral antibiotics (e.g. 2 h before the operation for ciprofloxacin tablets). A dose will be re-administered intraoperatively (4 h after the initial dose for cefazolin) when the duration of the intervention from initiation of preoperative dose is greater than twice the half-life of the drug agent. When blood loss is significant (≥ 1.5 L), an additional dose must be re-administered intraoperatively after fluid resuscitation. Postoperative doses are recommended for interventions specified in the guidelines, in the event of an intervention with a higher risk of postoperative infection (long, complex intervention) or if a postoperative infection would have serious consequences (implanted prosthetic material)
Duration of prophylaxis	The antibiotic prophylaxis should not exceed 24 h in total (except in heart valve replacement surgery where 48 h of prophylaxis should not be exceeded)

Prophylactic antibiotic regimens recommended in the updated institutional guidelines for the operations included in the study are:

- for hip prosthesis and coronary artery bypass graft surgery:
  o cefazolin i.v (+ vancomycin i.v if meticillin-resistant Staphylococcus aureus (MRSA) carriage)
  p vancomycin i.v + aztreonam i.v for patients with penicillin IgE-mediated allergy
- for colorectal surgery:
  o cefazolin i.v + metronidazole i.v (+ vancomycin i.v if MRSA carriage)
  p vancomycin i.v + aztreonam i.v + metronidazole i.v for patients with penicillin IgE-mediated allergy
- for transurethral resection of the prostate:
  o ciprofloxacin oral
  p cefuroxim i.v for patients with ciprofloxacin IgE-mediated allergy
- for endoscopic retrograde cholangiopancreaticography and in case of biliary obstruction (cholestasis):
  o amoxicillin–clavulanate i.v
  p moxifloxacin i.v for patients with penicillin IgE-mediated allergy

### Audits and stewardship interventions

Collaborative physician-pharmacist combined interventions started after the 2016 baseline period and included: (i) From November to December 2016, the central role of a pharmacist in the antibiotic stewardship multidisciplinary team for compilation of updated guidelines, audits, feedback of audit and educational seminar to prescribing physicians; (ii) From January 9, 2017 to April 21, 2017 (test period), the pharmacist aiming to implement the updated guidelines by making outreach visits to practitioners and delivering pre-operatively nominative kits containing the antibiotics with a written recommendation adapted to the type of intervention and to patient factors (recommendation also made available in the electronic patient record); (iii) In the period May–August 2017, the pharmacist making audits and feedback to the stewardship team; (iv) A physician-pharmacist collaboration developing an internal computer-based decision tool (https://db.serv-idb.net/antibioproph) validated by the Management Group of Antibiotics and diffused in the hospital in December 2018 (use recommended but not mandatory); (v) In the period July 2019-February 2020, the pharmacist making audits and feedback to the Management Group of Antibiotics and to prescribing physicians; (vi) In the period September–November 2021, the pharmacist finalizing audits.

### Data collection and statistical analysis

For each group of the study, data were collected from patients’ medical records. Compliance to guidelines was evaluated within each group through the seven items described here above.

In order to identify risk factors associated with non-compliance, a retrospective observational transversal study was achieved on the baseline group. In this study, the outcome variables were the compliance rates to guidelines in terms of the seven items audited and the independent variables were as follows: age, obesity, gender, IgE mediated penicillin (or Ciprofloxacin) allergy, multidrug-resistant organisms, ASA Score > 2, length of preoperative stay, type of intervention, surgeon or gastroenterologist, anesthetist, presence of a nurse anesthetist during the intervention, duration of the intervention, blood loss during surgery ≥ 1.5L. A multivariate statistical analysis (Wald test) was thus realized considering variables with a *P* < 0.10 in the univariate analysis. All the tests were bilateral, and the significance level for p-values was 0.05. The overall significance of the model was determined using the *χ*^2^ test at a significance level of 0.05. Interventions for which the studied outcome variable was missing were excluded (3 excluded for the compliance rate in terms of "*Time of pre-operative dose administration*", 2 excluded for the compliance rate in terms of "*Duration of prophylaxis*"). Interventions for which the independent variables studied was missing were also excluded (only 1 excluded for the dependent variable "*ASA score* > *2*").

Odds ratios for the relationships between each independent variable and each outcome variable were then determined.

The groups from year to year were then compared in terms of clinical and demographic characteristics and in terms of compliance to guidelines for each of the seven items audited. Data were analyzed using *χ*^2^ for categorical data (sex, number of patients per type of intervention, number of long duration interventions (> 3 h), number of allergic patients, compliance to guidelines for each of the 7 items audited) and *t* tests for continuous data (age).

Data were entered and subsequently analyzed using Microsoft Excel (version 2016; Microsoft Corporation, Redmond, WA, USA) except for the multivariate statistical analysis that was performed on the programming software *R* (*R* 3.2.3, December 2015, *R* Core Team). The missing data corresponding to an outcome variable were excluded from the statistical analysis (for the variable "*Compliance rate in terms of time of pre-operative dose administration*": 3, 10, 1, 3, 4 and 5 data were excluded from the baseline group, the test group, the post-test group, the post-computerized tool group, the first group of the COVID-19 period and the second group of the COVID-19 period, respectively; for the variable "*Compliance rate in terms of duration of prophylaxis*": 2 data were excluded from the baseline group and 2 from the second group of the COVID-19 period).

Data were analyzed using a t test for comparison between the baseline group and the test group in terms of prophylactic antibiotics cost.

## Results

In total, 760 interventions were recorded within the six groups described in Table 2. The groups were constituted by using identical time period to eliminate any potential seasonal influence.

**Table2:** Clinical and demographic characteristics of the six audited groups

Characteristics	Baseline group PERIOD 0	Test Group PERIOD 1	Post-test group PERIOD 2	Post-computerized tool group PERIOD 3	First group of the COVID-19 period PERIOD 4	Second group of the COVID-19 period PERIOD 5	P ^(a)^	P ^(b)^	P ^(c)^	P ^(d)^	P ^(e)^	P ^(f)^
							^(a)^ *Comparing the baseline group with the test group*	^(b)^ *Comparing the test group with the post-test group*	^(c)^ *Comparing the post-test group with the post-computerized tool group*	^(d)^ *Comparing the post-computerized tool group with the first group of the COVID-19 period*	^(e)^ *Comparing the first group of the COVID-19 period with the second group of the COVID-19 period*	^(f)^ *Comparing the second group of the COVID-19 period with the post-computerized tool group*
Number of Interventions, *n*	130	118	124	120	116	152						
Female, *n* (%)	48 (36.9)	49 (41.5)	57 (46)	34 (28.3)	53 (46)	57 (37.5)	0.46	0.49	0.004*	0.006*	0.18	0.11
Age (yr), mean ± SD	66.3 ± 11.7	68.4 ± 13.8	65.8 ± 13.6	65 ± 11.1	65.7 ± 13.7	66.7 ± 14.8	0.21	0.15	0.62	0.67	0.57	0.28
Transurethral resection of the prostate, *n* (%)	26 (20)	11 (9.3)	9 (7.3)	12 (10)	4 (3.5)	11 (7.2)	0.02*	0.56	0.45	0.045*	0.18	0.42
Coronary artery bypass grafting, *n* (%)	38 (29.2)	34 (28.8)	30 (24.2)	46 (38.3)	32 (27.6)	57 (37.5)	0.94	0.42	0.02*	0.08	0.09	0.89
Colorectal surgery, *n* (%)	17 (13.1)	22 (18.6)	19 (15.3)	12 (10)	23 (19.8)	24 (15.8)	0.23	0.49	0.21	0.03*	0.39	0.16
Hip prosthesis, *n* (%)	30 (23.1)	34 (28.8)	38 (30.7)	31 (25.8)	35 (30.2)	32 (21.1)	0.30	0.76	0.40	0.46	0.09	0.35
Endoscopic retrograde cholangiopancreatography, *n* (%)	19 (14.6)	17 (14.4)	28 (22.6)	19 (15.8)	22 (19)	28 (18.4)	0.96	0.10	0.18	0.53	0.91	0.58
Antibiotic prophylaxis, *n* (%)	113 (86.9)	109 (92.4)	104 (83.9)	107 (89.2)	98 (84.5)	124 (81.6)	0.16	0.04*	0.23	0.29	0.53	0.08
Duration of interventio*n* > 3 h, *n* (%)	48 (36.9)	52 (44.1)	48 (38.7)	55 (45.8)	45 (38.8)	63 (41.5)	0.25	0.40	0.26	0.27	0.66	0.47
IgE Mediated Penicillin Allergy, n (%)	6 (4.6)	6 (5.1)	11 (8.9)	6 (5)	11 (9.5)	13 (8.6)	0.86	0.25	0.24	0.18	0.79	0.25

^*^significant

### Identification of risk factors of non-compliance in the baseline group (Period 0)

The baseline group included 130 interventions carried out between January 11, 2016 and April 22, 2016, as indicated in Table 2. The results of compliance to updated guidelines within the baseline group and the results of the multivariate statistical analysis are shown, respectively, in Tables 3 and 4. The following variables appeared as risk factors of non-compliance in the multivariate statistical analysis (overall significance of the model: *P* ≤ 0.0001): (i) in terms of indication: the variable *IgE-mediated penicillin allergy*; (ii) in terms of drug agent: the variables *IgE-mediated penicillin allergy*, *colorectal surgery* and *transurethral resection* of the prostate; (iii) in terms of dose: the variables *colorectal intervention* and *transurethral resection of the prostate*; (iv) in terms of route of administration: the variables *transurethral resection of the prostate* and *two anesthetists*; (v) in terms of time of pre-operative dose administration: the variable *transurethral resection of the prostate*; (vi) in terms of duration of prophylaxis: the variable *hip prosthesis*.

**Table 3:** Results of compliance to updated guidelines

	Compliance, *n* (%)						P ^(a)^	P ^(b)^	P ^(c)^	P ^(d)^	P ^(e)^	P ^(f)^
	Baseline group PERIOD 0	Test Group PERIOD 1	Post-test group PERIOD 2	Post-computerized tool group PERIOD 3	First group of the COVID-19 period PERIOD 4	Second group of the COVID-19 period PERIOD 5	Comparing the baseline group with the test group	Comparing the test group with the post-test group	Comparing the post-test group with the post-computerized tool group	Comparing the post-computerized tool group with the first group of the COVID-19 period	Comparing the first group of the COVID-19 period with the second group of the COVID-19 period	Comparing the second group of the COVID-19 period with the post-computerized tool group
Indication	125 (96.2)	118 (100)	121 (97.6)	118 (98.3)	108 (93.1)	148 (97.4)	0.03*	0.09	0.68	0.046*	0.09	0.09
Drug agent(s)	108 (83.1)	115 (97.5)	111 (89.5)	111 (92.5)	101 (87.1)	133 (87.5)	< 0.01*	0.01*	0.42	0.17	0.92	0.92
Dose(s)	106 (81.5)	115 (97.5)	111 (89.5)	105 (87.5)	98 (84.5)	126 (82.9)	< 0.01*	0.01*	0.62	0.50	0.73	0.73
Route of administration	113 (86.9)	117 (99.2)	118 (95.2)	115 (95.8)	106 (91.4)	137 (90.1)	< 0.01*	0.06	0.80	0.16	0.73	0.73
Time of pre-operative dose administration	98 (77.2)	99 (91.7)	97 (78.9)	88 (75.2)	86 (76.8)	117 (79.6)	< 0.01*	< 0.01*	0.50	0.78	0.59	0.59
Number of administration(s)	56 (43.1)	88 (74.6)	67 (54.0)	68 (56.7)	51 (44.0)	85 (55.9)	< 0.01*	< 0.01*	0.68	0.05	0.05	0.05
Duration of prophylaxis	107 (83.6)	114 (96.6)	105 (84.7)	108 (90)	101 (87.1)	136 (90.7)	< 0.01*	< 0.01*	0.21	0.48	0.35	0.35

^*^significant

**Table 4:** Risk factors of non-compliance to SAP guidelines identified in the baseline group

Risk factor of non Compliance	Compliance item impacted	*Z*-test	*P*	OR (95% IC)
**IgE Mediated Penicillin Allergy**	Indication	− 2.383	0.0172	0.0345(0.0022–0.5502)
	Drug agent(s)	− 2.012	0.0442	0.1282(0.0173–0.9481)
**Colorectal surgery**	Drug agent(s)	− 3.233	< 0.01	0.0187(0.0017–0.2086)
	Dose(s)	− 3.321	< 0.01	0.0623(0.0194–0.2007)
**Transurethral resection of the prostate**	Drug agent(s)	− 3.07	0.021	0.0933(0.0205–0.4243)
	Dose(s)	− 2.824	< 0.01	0.1614(0.0455–0.5724)
	Route of administration	− 4.44	< 0.01	0.0393(0.0094–0.1641)
	Time of pre-operative dose administration	− 6.093	< 0.01	0.0293(0.0094–0.0918)
**Hip prosthesis**	Duration of prophylaxis	− 5.002	< 0.01	0.0602(0.0200–0.1811)
**Anesthetist Y**	Route of administration	− 2.377	0.0174	0.0761 (0.0091–0.6365)
**Anesthetist Z**	Route of administration	− 2.074	0.0381	0.0815 (0.0076–0.8713)

### Improvement of SAP practices in the test group that received real time pharmaceutical stewardship (Period 1)

Regarding the test group, 118 surgical interventions were included between January 9, 2017 and April 21, 2017. In terms of clinical and demographic characteristics, the test group was similar to the pre-test group (Table 2). Compared with the pre-test group, the compliance was significantly increased in the test group for all the seven criteria audited (*p* < 0.05). Moreover, as requested by the BAPCOC, the items *drug agent(s)* (97.5%) and *duration of prophylaxis* (96.6%) became, in the test group, compliant to local guidelines in more than 90% of cases.

### No economic impact on antibiotic prophylaxis comparing the baseline group with the test group

The mean prophylactic antibiotics cost (mean ± standard deviation) for the patients in the baseline group was 9.2 ± 6.8 € while it was 10.8 ± 10.9 € for the patients in the test group. The statistical analysis did not show a significant difference (*p* = 0.17) between the two groups in terms of prophylactic antibiotics cost.

### Decrease of the compliance to guidelines in the post-test group (Period 2)

Between January 8, 2018 and April 20, 2018, 124 surgical interventions were recorded to constitute the post-test group (Table 2). The comparison of antibiotic prophylaxis practices in the 2017 test group (*n* = 118) versus the 2018 post-test group (*n* = 124).

revealed a significant decrease in compliance for 5 of the 7 items assessed (*p* < 0.05 for the items *Drug agent(s), Dose(s), Time of pre-operative dose administration, Number of administration(s),* and *Duration of prophylaxis*). The rates of compliance in terms of drug agent(s) (89.5%) and duration of prophylaxis (84.7%) had thus fallen back below 90% in the post-test group.

### Non-statistically significant impact of the computerized decision support tool in terms of compliance to guidelines (Period 3)

The post-computerized tool group was constituted between January 7, 2019 and April 19, 2019 and included 120 surgical interventions (Table 2). The comparison of antibiotic prophylaxis practices in the 2018 post-test group (*n* = 124) versus the 2019 post-computerized tool group (*n* = 120) revealed a trend of compliance increase for 5 of the 7 items assessed (non-significant, *p* > 0.05) allowing a return above 90% for the two BAPCOC indicators (the items *drug agent(s)* (92.5%) and *duration of prophylaxis* (90%)).

### No obvious impact of the COVID-19 pandemic in terms of compliance to guidelines (Period 4 & 5)

The first group of the COVID-19 period was similar to the 2019 post-computerized tool group in terms of demographics characteristics (*p* > 0.05) except for the gender (*p* < 0.05); regarding clinical characteristics, statistically significant differences (*p* < 0.05) were observed for certain types of interventions decrease in the rate of transurethral resections of the prostate and increase in the rate of colorectal procedures, *p* < 0.05 (Table 2). Therefore, a bias, probably linked to the first wave of COVID-19 in Belgium, could not be excluded when comparing the compliance rates between these two groups of 2019 and 2020. Nevertheless, there were no statistically significant differences for all items of compliance except for the variable *indication of prophylaxis* (*p* < 0.05). The two groups of the COVID-19 period were, for their part, similar with respect to demographics and clinical characteristics and in terms of compliance rates. A similarity was also observed when comparing demographics, clinical and compliance characteristics of the second group of the COVID-19 period with the 2019 post-computerized tool group (Tables 2 and 3).

## Discussion

At the beginning of this work, surgical antibiotic prophylaxis practices were not in line with updated national and international guidelines. Indeed, the first audit, achieved on the baseline group, revealed an over 13% non-compliance to updated guidelines for six of the seven items audited. Through a multivariate statistical analysis, the present work allowed to formulate hypotheses regarding the various risk factors associated with this non-compliance: Penicillin IgE-mediated allergy, certain types of surgery (colorectal surgery, hip prosthesis surgery, transurethral resection of the prostate) and two anesthesiologists who were frequently associated with transurethral resections of the prostate. Therefore, we cannot exclude some dependence between these two practitioners and the transurethral resections of the prostate.

These data are consistent with those of the literature which also revealed as risk factors for non-compliance IgE-mediated penicillin allergy and certain types of surgery, in particular urological surgery and digestive surgery [7, 23].

As reported by Muller's team for the pre-operative administrations [7], one of the least respected criteria in the baseline group was the time of pre-operative dose administration. Post-operatively, the number of administrations was frequently non-compliant with the main reason being a number of administrations higher than that recommended. Lack of education and incomplete professional rules were the main barriers associated with the risk factors identified in our study. Our former institutional guidelines in particular, did not specify intraoperative re-administration and alternative drugs in the event of IgE-mediated allergy.

After analysis confirming the similarity between groups, a statistical analysis indicated a significant difference in compliance between the test group and the baseline group for all audited items (Table 4). These results show a positive impact of our global stewardship strategy on compliance to the updated recommendations for surgical antibiotic prophylaxis. However, this improvement in compliance was not associated with an economic benefit since we observed no significant difference between the baseline group and the test group in terms of prophylactic antibiotics cost.

To our knowledge, the literature does not mention studies developing a global strategy of this order to improve SAP practices. Comparing with previous reports applying more restrained strategies, our results also confirm a positive impact on SAP practices, in particular with an increase in the rational selection of antibiotics, the appropriate duration of prophylaxis, and the correct time of administration of the first preoperative dose [15, 16, 19, 24]. Moreover, our global strategy herein developed allowed us to reach rates of compliance higher than those obtained in previous reports [15, 16, 19]. As described in the guidelines written by the Infectious Diseases Society of America [25], this type of strategy with a persuasive aim has certain advantages and disadvantages which were also observed during the implementation of the action plan in our hospital.

For the benefits encountered:

- Increased visibility of the stewardship program thanks to the presence of the pharmacist in the operating room and in departments involved in the study.
- Establishment of good collegial relationships facilitating consensus (in the various departments involved in the study, presentation of the pharmacist as the referent for "Antibioprophylaxis" with facilitation of collaboration and support to prescription).
- Educational benefit and improved adherence to recommendations by prescribers (combinations of persuasive interventions accomplished during the action plan).

For the inconveniences encountered.

- Success depends on the interventional method used. In this work, the dissemination/display of paper recommendations and the delivery by the pharmacist of antibiotic prophylaxis kits incorporating a recommendation sheet adapted to the patient were highly effective and largely contributed to the success of practices improvement.
- Intensive work and determination are required, especially for the delivery of the kits early in the morning for the very first surgeries and a constant monitoring of the operating schedule that can change from hour to hour.
- Need to convince practitioners who can be resistant to change.

After this test phase, we evaluated the sustained effect of the collaborative physician-pharmacist stewardship implemented. The results revealed that, without active stewardship, there were a significant decrease in the compliance to guidelines for 5 out of 7 items assessed (*p* < 0.05, comparing the test group with the post-test group). Despite this, for all items assessed, the rates of compliance in the post-test group were still higher than those measured in the baseline group.

The impact of computerized decision support tool was non significant, with similar compliances to updated guidelines between the post-test group and the post-computerized tool group (*p* > 0.05). There was however a slight increase in compliance for 5 out of 7 items assessed (*p* > 0.05) allowing a return above 90% in terms of compliance rate for the two BAPCOC indicators studied. In our hospital, the computerized decision support tool we implemented has also certain advantages and disadvantages.

For the benefits encountered:

- The tool integrates the guidelines updated and validated by the different actors of antibiotic prophylaxis and allows to consider specific patient criteria.
- The tool allows rapid and efficient decision-making, adapted to the patient's parameters and in compliance with guidelines.
- Recommendations are accessible via a computer link (also from outside the hospital).
- Stewardship strategy is less labor-intensive.
- The tool can sensitize the teams to the importance of antibiotic prophylaxis.

For the inconveniences encountered.

- No connection with the computerized record of the patient (requiring a manual encoding by practitioners).
- Absence of reminder recalling the pre-operative administration of antibiotics.
- Underused by practitioners (use not mandatory).

The above three points would need to be developed and implemented in order to positively impact SAP practices. As reported in the literature [16, 26–28], computer-based help for clinical decision and prescription seems to be a useful tool for surgical antibiotic prophylaxis but it should be accompanied by direct regular educational measures.

On 11 March 2020, the Belgian Hospital in which this study was conducted admitted for the first time a patient with a positive SARS-CoV-2 reverse transcriptase polymerase chain reaction (RT-PCR) test [20]. The Hospital & Transport Surge Capacity committee in Belgium announced that hospitals must stop all consultations, examinations and interventions planned from 14 March 2020. On April 30, 2020, the committee communicated guidelines to hospitals for a gradual resumption of regular care [29]. The first group of the COVID-19 period was therefore affected in terms of distribution of the different interventions studied, with a particular decrease in the rate of transurethral resections of the prostate. In this 2020 group, only compliance rates in terms of indication were significantly decreased compared to the 2019 group. The two groups of the COVID-19 period were, for their part, similar with respect to all demographic, clinical and compliance characteristics. In order to exclude a possible bias linked to the redistribution of interventions from the 2020 first wave of COVID-19, we also compared data collected for the second group of the COVID-19 period with those of the 2019 group. This comparison revealed no statistically significant differences in terms of demographic, clinical and compliance characteristics confirming that the COVID-19 pandemic did not affect the compliance of SAP practices to institutional guidelines. Out of 147 RT-PCR tests carried before hospitalization within the second group of the COVID-19 period, 5 were found to be positive with no obvious impact on compliance (data not shown).

The stewardship carried out in this work appears to be adapted to the objective of renewing antibiotic prophylaxis practices. However, more improvement measures are needed for a long-term effect. In particular, the preoperative computerized and automated prescription of SAP based on computerized patient data; the delivery by the pharmacy of nominative SAP kits based on the doctor's computerized prescription; the repetition of active interventions and audits in order to maintain the awareness of practitioners, particularly in a university hospital with a high turnover of doctors in training. These quality improvement initiatives require the dedication of specific personnel at all decision-making levels and the release of time dedicated to the maintenance and continuous improvement of quality. The support of the members of the hospital management is therefore essential.

The study we developed presents a series of limitations commonly encountered in observational and quasi-experimental studies. On one hand, confounding bias linked to the change of practioners from year to year cannot be ruled out in the quasi-experimental study. Also, in the observational study, some uninvestigated factors could influence noncompliance. For practical, economic and swiftness reasons, convenience sampling was selected; but, indeed, the absence of randomization may reveal a selection bias. Identical seasonal periods and the same period of time had been determined in order to limit the occurrence of this type of bias. On the other hand, the study could not be blinded; practitioners were aware of participating in a study. Biases such as the Hawthorne effect could therefore appear [30]. Also, our data collection procedure resulted in the absence of some data. Collecting a full amount of data prospectively would require such a large amount of work that the IT solution used for retrospective analysis was clearly necessary. The inclusion of several services in this study, aids the generalizability of the results, but the limited time and monocentric aspect of the study significantly reduce the number of patients within each unique category, thereby limiting the prediction power. The limited number of patients within each group and the lack of accessibility of patient health data after discharge from hospital did not allow to evaluate the evolution of clinical or microbiological outcomes such as incidence of surgical site infections or infections with antimicrobial-resistant bacteria. Despite this, the compliance with SAP recommendations that we measured in this work represents an undeniable quality indicator in the prevention process.

## Conclusions

Rational use of SAP requires a long-term proactive, collaborative and common approach including SAP prescribers and antibiotic stewardship multidisciplinary team. Indeed, this study shows that, among all stewardship strategies implemented to positively impact SAP practices, the most performant strategy clearly appear to be real-time pharmaceutical interventions in the operating area and in the care units concerned by antibiotic prophylaxis. The discontinuation of these active interventions, however, results in a slight decrease in compliance to local guidelines. Finally, SAP practices did not appear to be affected by the COVID-19 pandemic in terms of compliance to guidelines.

## Data Availability

The datasets used and/or analysed during the current the study are available from the corresponding author on reasonable request.

## Abbreviations

ASA: American society of anesthesiologists
SSI: Surgical site infection
SAP: Surgical antibiotic prophylaxis
BAPCOC: Belgian antibiotic policy coordination committee
COVID-19: Coronavirus 2019 disease
CABG: Coronary artery bypass grafting
ERCP: Endoscopic retrograde cholangiopancreaticography

## References

[1] Nepogodiev D (2019). Global burden of postoperative death. Lancet.

[2] Dony P, Pirson M, Boogaerts JG (2018). In- and out-hospital mortality rate in surgical patients. Acta Chir Belg.

[3] Dony P (2019). Anaesthesia care team improves outcomes in surgical patients compared with solo anaesthesiologist: An observational study. Eur J Anaesthesiol.

[4] National Institute for Health and Care Excellence. Surgical site infection [NICE Quality standard QS49]. 2013. www.nice.org.uk/guidance/qs49.

[5] National Institute for Health and Care Excellence. Surgical site infections: prevention and treatment [NICE guideline NG125]. 2019. www.nice.org.uk/guidance/ng125. 31909935

[6] Hall C, Allen J, Barlow G (2015). Antibiotic prophylaxis. Surg - Oxford Int Edition.

[7] Muller A (2015). Surgical antibiotic prophylaxis compliance in a university hospital. Anaesthesia, Critical Care & Pain Medicine.

[8] National Institute for Health and Care Excellence. Clostridium difficile infection: risk with broad-spectrum antibiotics [NICE Evidence summary ESMPB1]. 2015. www.nice.org.uk/guidance/esmpb1.

[9] Balligand E, M Costers, and E Van Gastel, Belgian Antibiotic Policy Coordination Committee - Note de politique pour la législature 2014–2019. BAPCOC, 2014.

[10] Ristić S (2010). Are local clinical guidelines useful in promoting rational use of antibiotic prophylaxis in Caesarean delivery?. Pharm World Sci.

[11] Dellit TH (2007). Infectious diseases society of America and the Society for healthcare epidemiology of America guidelines for developing an institutional program to enhance antimicrobial stewardship. Clin Infect Dis.

[12] Lallemand S (2002). Evaluation of practices in surgical antimicrobial prophylaxis in the Franche-Comté before and after implementation of an information program. Annales Françaises d'anesthésie et de réanimation.

[13] D’Escrivan T (2005). Antibioprophylaxie chirurgicale : adéquation aux recommandations et impact d’une action d’information ciblée. Annales Françaises d'Anesthésie et de Réanimation.

[14] Gindre S (2004). Antibioprophylaxie chirurgicale : évaluation de l’application des recommandations et validation des kits d’antibioprophylaxie. Annales Françaises d'Anesthésie et de Réanimation.

[15] Prado MAMB (2002). The implementation of a surgical antibiotic prophylaxis program: The pivotal contribution of the hospital pharmacy. Am J Infect Control.

[16] Fayolle-Pivot L (2013). Contribution of information technologies to assess and improve professional practice: Example of management of surgical antibiotic prophylaxis. Annales Françaises d’Anesthésie et de Réanimation.

[17] Zhang H-X (2014). Pharmacist interventions for prophylactic antibiotic use in urological inpatients undergoing clean or clean-contaminated operations in a chinese hospital. PLoS ONE.

[18] Zhou Y (2015). Impact of pharmacist intervention on antibiotic use and prophylactic antibiotic use in urology clean operations. J Clin Pharm Ther.

[19] Zhou L (2016). Optimizing prophylactic antibiotic practice for cardiothoracic surgery by pharmacists’ effects. Medicine.

[20] Milas S, Antibiotic use in patients with Coronavirus disease, (2019). (COVID-19): outcomes and associated factors. Acta Clin Belg.

[21] Sanford J, et al, The Sanford guide to antimicrobial therapy: The Belgian/Luxembourg Version 2012–2013. ed. 2013.

[22] Bratzler D (2013). Clinical practice guidelines for antimicrobial prophylaxis in surgery. Am J Health Syst Pharm.

[23] Simon A-M (2012). Facteurs prédictifs de non-conformité d’antibioprophylaxie chirurgicale au cours d’un audit clinique prospectif. Annales françaises d'anesthésie et de réanimation.

[24] Segala F et al, Antibiotic appropriateness and adherence to local guidelines in perioperative 10.1186/s13756-020-00814-6 PMC7586646 33106190

[25] prophylaxis: results from an antimicrobial stewardship intervention. Antimicrobial resistance and infection control, 2020. **9**: p. 164. 10.1186/s13756-020-00814-6 PMC7586646 33106190

[26] Barlam TF (2016). Implementing an antibiotic stewardship program: guidelines by the infectious diseases society of America and the society for healthcare epidemiology of America. Clinic Infectious Diseases: Official Publ Infectious Diseases Soc Am.

[27] Nair BG (2011). Automated electronic reminders to improve redosing of antibiotics during surgical cases: Comparison of two approaches. Surg Infect.

[28] Nair BG (2010). Feedback mechanisms including real-time electronic alerts to achieve near 100% timely prophylactic antibiotic administration in surgical cases. Anesth Analg.

[29] Wax DB (2007). The effect of an interactive visual reminder in an Anesthesia information management system on timeliness of prophylactic antibiotic administration. Anesth Analg.

[30] VandeVoorde C et al, Gestion de la capacité hospitalière en Belgique durant la première vague de la pandémie de COVID-19, in KCE Report 335Bs. 2020.

[31] McCambridge J (2014). Systematic review of the Hawthorne effect: New concepts are needed to study research participation effects. J Clin Epidemiol.

